# Polio outbreak response, Yemen

**DOI:** 10.2471/BLT.23.290122

**Published:** 2023-10-12

**Authors:** Ibrahem Abduallah Beshr, Mohammed Sadeq Beshr, Hibah Abdulqader Al-Qubati

**Affiliations:** a Faculty of Medicine and Health Sciences, Sana’a University, Wadi Dhaher Road, PO Box 13078, Sana’a, Yemen.

## Abstract

**Problem:**

A decrease in vaccine coverage in conflict-affected areas has placed Yemen at higher risk of polio outbreaks caused by vaccine-derived poliovirus strains.

**Approach:**

In response to polio outbreaks, the Yemeni health ministry and partners initiated multiple vaccination campaigns to deliver vaccines to children. We also implemented several measures to enhance communication, education, health promotion and hygiene, especially in camps for internally displaced people.

**Local setting:**

In 2009, Yemen achieved polio-free status and maintained it until 2019. However, the ongoing political conflict since 2015, coupled with challenges in delivering the polio vaccine to conflict-affected areas, resulted in two polio outbreaks: 35 cases caused by vaccine-derived poliovirus strain 1 between 2019 and 2021, and 230 cases due to vaccine-derived poliovirus strain 2 between November 2021 and December 2022.

**Relevant changes:**

In response to the first outbreak, by the end of 2020, we vaccinated 7.2 million children through nationwide vaccination campaigns, except in Sa’ada governorate due to a ban by the authorities. By the end of 2021, 3 800 313 children younger than 5 years had received polio vaccines. For the second outbreak, by the end of 2022, 4 463 389 vaccines had been given to children younger than 10 years, and 1 217 423 to those younger than 5 years.

**Lessons learnt:**

Vaccination campaigns in conflict-affected areas with low vaccine coverage remain crucial in eradicating polio. Efforts are needed to reach vulnerable groups such as displaced populations. Advocacy, communication and social mobilization actions help ensure broader public inclusion and participation in vaccination efforts to prevent polio outbreaks.

## Résumé

**Problème:**

Avec le déclin de la couverture vaccinale dans les régions en conflit, le Yémen encourt un risque accru d'épidémies de poliomyélite dues à un poliovirus dérivé d'une souche vaccinale.

**Approche:**

Face aux épidémies de poliomyélite, le Ministère de la Santé yéménite et ses partenaires ont lancé plusieurs campagnes de vaccination des enfants. Nous avons également déployé de nombreuses mesures visant à améliorer la communication, l'éducation, la promotion de la santé et l'hygiène, en particulier dans les camps abritant des personnes déplacées à l’intérieur du pays.

**Environnement local:**

En 2009, le Yémen avait été déclaré exempt de poliomyélite et a conservé ce statut jusqu'en 2019. Toutefois, les luttes politiques qui font rage depuis 2015, associées aux difficultés rencontrées dans la fourniture du vaccin antipoliomyélitique dans les régions en conflit, ont entraîné deux épidémies: 35 cas ont été causés par un poliovirus dérivé d'une souche vaccinale de type 1 entre 2019 et 2021, et 230 cas par un poliovirus dérivé d'une souche vaccinale de type 2 entre novembre 2021 et décembre 2022.

**Changements significatifs:**

Pour lutter contre la première épidémie, fin 2020, nous avons vacciné 7,2 millions d'enfants dans le cadre de campagnes de vaccination à l'échelle nationale, à l'exception du gouvernorat de Saada, en raison de l'interdiction imposée par les autorités. Fin 2021, 3 800 313 enfants de moins de cinq ans avaient reçu leur vaccin antipoliomyélitique. Lors de la seconde épidémie, fin 2022, 4 463 389 vaccins avaient été administrés aux enfants de moins de 10 ans et 1 217 423 aux enfants de moins de 5 ans.

**Leçons tirées:**

Les campagnes de vaccination dans les régions en conflit où la couverture vaccinale est faible demeurent cruciales pour l'éradication de la poliomyélite. Des efforts sont nécessaires pour atteindre les groupes vulnérables tels que les populations déplacées. Les actions de sensibilisation, de communication et de mobilisation sociale permettent de toucher un plus large public et d'encourager la participation aux efforts de vaccination, essentiels dans la prévention des épidémies de poliomyélite.

## Resumen

**Situación:**

La disminución de la cobertura de vacunación en las regiones afectadas como consecuencia del conflicto ha hecho que Yemen corra un mayor riesgo de brotes de poliomielitis causados por cepas de poliovirus derivadas de la vacuna.

**Enfoque:**

En respuesta a los brotes de poliomielitis, el Ministerio de Salud de Yemen y sus asociados emprendieron múltiples campañas de vacunación para suministrar vacunas a los niños. También aplicamos varias medidas para mejorar la comunicación, la educación, la promoción de la salud y la higiene, especialmente en los campamentos para desplazados internos.

**Marco regional:**

En 2009, Yemen logró la condición de país libre de poliomielitis y la mantuvo hasta 2019. Sin embargo, el conflicto político en curso desde 2015, junto con las dificultades para suministrar la vacuna antipoliomielítica a las regiones afectadas por el conflicto, dieron lugar a dos brotes de poliomielitis: 35 casos causados por la cepa 1 del poliovirus derivado de la vacuna entre 2019 y 2021, y 230 casos debidos a la cepa 2 del poliovirus derivado de la vacuna entre noviembre de 2021 y diciembre de 2022.

**Cambios importantes:**

En respuesta al primer brote, a finales de 2020 habíamos vacunado a 7,2 millones de niños a través de campañas de vacunación en todo el país, excepto en la gobernación de Saada debido a una prohibición de las autoridades. A finales de 2021, 3 800 313 niños menores de 5 años habían sido vacunados contra la poliomielitis. Para el segundo brote, a finales de 2022 se habían administrado 4 463 389 vacunas a niños menores de 10 años y 1 217 423 a menores de 5 años.

**Lecciones aprendidas:**

Las campañas de vacunación en regiones afectadas por conflictos con baja cobertura de vacunación siguen siendo esenciales para erradicar la poliomielitis. Se necesitan esfuerzos para llegar a los grupos vulnerables, como las poblaciones desplazadas. Las acciones de promoción, comunicación y movilización social ayudan a garantizar una mayor inclusión y participación del público en los esfuerzos de vacunación para prevenir los brotes de poliomielitis.

## ملخص

**المشكلة:**

إن الانخفاض في تغطية اللقاحات للمناطق المتضررة من النزاع، أدى لوضع اليمن في موقع أكثر خطورة لتفشي مرض شلل الأطفال الناتج عن سلالات فيروس شلل الأطفال المشتقة من اللقاح.

**الأسلوب:**

استجابةً لحالات تفشي مرض شلل الأطفال، أطلقت وزارة الصحة اليمنية وشركاؤها حملات تطعيم متعددة لتوصيل اللقاحات للأطفال. كما قمنا بتنفيذ العديد من التدابير لتعزيز التواصل، والتعليم، والارتقاء بالصحة والنظافة، وخاصة في مخيمات النازحين بالداخل.

**المواقع المحلية:**

في عام 2009، حقق اليمن حالة الخلو من مرض شلل الأطفال، وحافظ عليها حتى عام 2019. ومع ذلك، أدى الصراع السياسي الدائر منذ عام 2015، مقترنًا بالتحديات في توصيل لقاح شلل الأطفال إلى المناطق المتضررة من النزاع، إلى حالتين من تفشي مرض شلل الأطفال: 35 حالة ناجمة عن سلالة فيروس شلل الأطفال المشتقة من اللقاح 1 بين عامي 2019 و2021، و230 حالة بسبب سلالة فيروس شلل الأطفال المشتقة من اللقاح 2 بين نوفمبر/تشرين ثاني 2021 وديسمبر/كانون أول 2022.

**التغيّرات ذات الصلة:**

استجابةً لحالة التفشي الأولى، بنهاية عام 2020، قمنا بتطعيم 7.2 مليون طفل من خلال حملات التطعيم المنتشرة على مستوى الدولة، باستثناء محافظة صعدة بسبب الحظر الذي فرضته السلطات. وبنهاية عام 2021، تلقى عدد 3800313 طفلاً تقل أعمارهم عن 5 سنوات لقاحات شلل الأطفال. وبالنسبة لحالة التفشي الثانية، بنهاية عام 2022، تم إعطاء 4463389 لقاحًا للأطفال الذين تقل أعمارهم عن 10 سنوات، و1217423 لقاحًا للأطفال الذين تقل أعمارهم عن 5 سنوات.

**الدروس المستفادة:**

إن حملات التطعيم في المناطق المتضررة من النزاع، والتي تعاني من تغطية منخفضة من اللقاحات، تظل ذات أهمية بالغة في استئصال مرض شلل الأطفال. هناك حاجة إلى بذل جهود للوصول إلى المجموعات المهمشة مثل السكان النازحين. وتساعد إجراءات الدعم، والاتصال، والتعبئة الاجتماعية على ضمان الإدماج والمشاركة العامة على نطاق أوسع في جهود التطعيم لمنع حالات تفشي مرض شلل الأطفال.

## 摘要

**问题:**

受冲突影响地区的疫苗覆盖率下降，使也门面临较高的脊髓灰质炎爆发风险。该脊髓灰质炎是由疫苗衍生脊髓灰质炎病毒株引起的。

**方法:**

为了应对脊髓灰质炎爆发，也门卫生部及其合作伙伴发起了多项为儿童接种疫苗的疫苗接种运动。我们还实施了若干措施，以加强沟通、宣传教育、促进健康和卫生条件，尤其针对境内流离失所者所居的营地。

**当地状况:**

2009 年，也门实现了“无脊髓灰质炎状态”，并一直维持此状态到 2019 年。然而，自 2015 年以来，持续的政治冲突，加上在向受冲突影响地区提供脊髓灰质炎疫苗方面面临的挑战，导致了两次脊髓灰质炎的爆发：2019 年至 2021 年期间，疫苗衍生脊髓灰质炎病毒株 1 型引起了 35 例脊髓灰质炎， 2021 年 11 月至 2022 年 12 月期间，疫苗衍生脊髓灰质炎病毒株 2 型引起了 230 例脊髓灰质炎。

**相关变化:**

为应对第一次脊髓灰质炎爆发，截至 2020 年底，除受当局禁令影响的萨达省外，我们通过全国范围内的疫苗接种运动为 720 万名儿童接种了疫苗。截至 2021 年底，已有 3,800,313 名 5 岁以下的儿童接种了脊髓灰质炎疫苗。为应对第二次爆发，截至 2022 年底，我们已为 10 岁以下儿童接种了 4,463,389 支疫苗，为 5 岁以下儿童接种了 1,217,423 支疫苗。

**经验教训:**

在受冲突影响、疫苗覆盖率低的地区开展疫苗接种运动对于根除脊髓灰质炎仍然至关重要。需要采取行动来帮助流离失所者等弱势群体。进行宣传、沟通和社会动员活动有助于确保更多的公众加入和参与预防脊髓灰质炎爆发的疫苗接种工作。

## Резюме

**Проблема:**

Снижение уровня охвата вакцинацией в зонах конфликта привело к тому, что в Йемене повысился риск возникновения вспышек полиомиелита, вызванных штаммами вакцинного полиовируса.

**Подход:**

В ответ на вспышки полиомиелита Министерство здравоохранения Йемена и партнеры провели многочисленные кампании по вакцинации детей. Также был реализован ряд мер по улучшению коммуникации, образования, укреплению здоровья и соблюдению гигиены, особенно в лагерях для перемещенных лиц.

**Местные условия:**

В 2009 году Йемен получил статус страны, свободной от полиомиелита, и сохранял его до 2019 года. Однако продолжающийся с 2015 года политический конфликт в совокупности со сложностями при доставке вакцины против полиомиелита в охваченные конфликтом районы привел к двум вспышкам полиомиелита: 35 случаев, вызванных штаммом 1 вакцинного полиовируса в период с 2019 по 2021 год, и 230 случаев, вызванных штаммом 2 вакцинного полиовируса в период с ноября 2021 года по декабрь 2022 года.

**Осуществленные перемены:**

В ответ на первую вспышку к концу 2020 года была проведена вакцинация 7,2 млн детей в рамках общенациональных кампаний по вакцинации, за исключением провинции Саада из-за запрета властей. К концу 2021 года вакцины против полиомиелита получили 3 800 313 детей в возрасте до 5 лет. Что касается второй вспышки, к концу 2022 года детям в возрасте до 10 лет было введено 4 463 389 вакцин, а детям в возрасте до 5 лет – 1 217 423.

**Выводы:**

Кампании по вакцинации в зонах конфликта с низким уровнем охвата вакцинацией по-прежнему имеют решающее значение для ликвидации полиомиелита. Необходимы усилия по охвату уязвимых групп населения, таких как перемещенные лица. Мероприятия по информационно-просветительской деятельности, коммуникации и социальной мобилизации способствуют более широкому вовлечению и участию населения в вакцинации с целью предотвращения вспышек полиомиелита.

## Introduction

In 2023, Yemen is facing a threat of polio outbreaks that could paralyse more children in a country already devastated by conflict, poverty, hunger and disease. Both mass vaccination and surveillance systems have been used in the past to achieve a polio-free status. In 1998, the World Health Organization (WHO) helped launch the acute flaccid paralysis surveillance system, with the aim of quickly identifying cases and collecting samples. This initiative was accompanied by mass vaccination campaigns throughout Yemen, as part of a concerted effort to eradicate polio.^1^ Yemen achieved polio-free status in 2009, and remained free of the disease until 2019. The current political conflict and war since 2015 has placed the country at a higher risk of polio and other vaccine-preventable outbreaks.

The oral polio vaccine contains three types of attenuated poliovirus strains (types 1, 2 and 3) that help people become immune to the disease. Sometimes, the attenuated virus in the vaccine can turn into a harmful strain called vaccine-derived poliovirus when not enough people get the vaccine. Vaccine-derived polioviruses can then be passed to other children through faeces and the ingestion of contaminated food and water.^2^ In areas with low immunization coverage, the virus can circulate for a long time and undergo genetic changes that make it more virulent and capable of causing paralysis. This is how outbreaks of polio can emerge in polio-free countries.^3^^,^^4^

Despite the ongoing conflict, Yemen still maintains a functioning and responsive acute flaccid paralysis surveillance system.^5^ However, the conflict has made it difficult for health workers to reach all children for polio vaccination, particularly in remote and conflict-affected areas. In some districts in the northern governorates, all vaccination activities for polio and other vaccine-preventable infectious diseases have been banned by the local authorities.^6^ A study of the impact of the war on vaccination coverage demonstrated a downward trend, especially in areas where there is armed conflict.^7^ The problem is compounded by poor sanitation and hygiene conditions in conflict-affected areas and camps for displaced people in Yemen, posing a significant risk of outbreaks of polio and other infectious diseases.^8^

## Local setting

Between 2019 and 2021, Yemen reported a total of 35 cases of polio caused by the vaccine-derived poliovirus strain 1. The first case occurred in 2019, followed by 31 cases in 2020, and three cases in 2021.^9^ The epicentre of the outbreak was in Sa’ada governorate, an area where door-to-door vaccinations had not been allowed by the northern authorities for several years.^9^

A second outbreak with a different polio strain (vaccine-derived poliovirus strain 2) occurred from November 2021 until December 2022. The total number of cases in this outbreak was 230, according to the Yemeni health ministry. Out of these cases, 197 were in the northern governorate, according to the Global Polio Eradication Initiative records.^10^ The vaccine-derived poliovirus strain 2 was identified in several governorates, including Aden, Marib, Sa’ada and Taiz.^9^ The total number of vaccine-derived poliovirus strain 2 cases in Yemen accounted for one third of the 634 global polio outbreaks in 2022.^11^

## Approach

In July 2020, the Yemeni health ministry, along with the Global Polio Eradication Initiative, started on-the-ground surveillance and implementation of prompt response measures to the polio outbreaks. The measures included national and sub-national immunization campaigns using the oral polio vaccine, and environmental surveillance to monitor the circulation of polioviruses. The global initiative partners, including WHO and the United Nations Children’s Fund (UNICEF), are working to overcome the challenges posed by insecurity, access restrictions, population movements, the pandemic of coronavirus disease 2019 (COVID-19) and the public’s reluctance to be vaccinated.^10^^,^^12^

In response to the first outbreak, we conducted two vaccination campaigns. The first round took place in July 2020, covering 10 governorates. The second round was in November 2020 in the north, and December 2020 in the south. For the second outbreak, three campaigns were initiated in the 12 southern governorates. The first two rounds took place in February and March 2022, while the third campaign occurred in May. In March 2023, the first round of the polio campaign was delivered in the southern governorates; however, in the northern governorates, where 65% of the population resides, vaccination outside of health facilities is still suspended by the northern authorities.

We used many strategies at every level to reach the target of vaccinating all children younger than 5 years. UNICEF, WHO and their partners implemented advocacy, communication and social mobilization actions nationwide several days before the start of the polio vaccination campaign. These actions were designed to sensitize and educate the public, raise awareness, reduce vaccine hesitancy, and to ensure broader public inclusion and participation.^6^^,^^12^^,^^13^ The advocacy, communication and social mobilization actions included meetings with representatives of government and the community, and religious leaders. Religious leaders held sessions at mosques, community meetings, women’s gatherings and schools. These sessions helped to promote polio vaccination, hygiene awareness and sanitation practices. We also used vehicles with megaphones, as well as numerous posters and banners at strategically placed locations.^6^^,^^12^^,^^13^ To enhance communication and support, community volunteers created mobile phone message groups (WhatsApp Messenger, Meta Platforms, Menlo Park, United States of America) to help with communication and support. The health ministry and UNICEF set up dedicated telephone numbers to allow the public to connect with health-care professionals with enquiries about polio disease, vaccines and various health-related topics.^6^^,^^12^^,^^13^

We also implemented the water, sanitation and hygiene programme to improve water safety. The programme staff delivered gender-specific latrines and hygiene kits to camps for internally displaced people, which is the most vulnerable population. They also contributed to the rehabilitation and maintenance of sanitation systems across the country. Furthermore, they aided in the repair of wastewater treatment plants. In terms of safe water supply, the programme staff helped deliver safe drinking water to camps of internally displaced people; and expanded water, sanitation and hygiene services to both rural and urban areas. Staff provided chlorination tablets, cleaning kits and water filters to camps and households as well. Additionally, the staff assisted in the rehabilitation and maintenance of multiple water collection points and systems (pumping stations and wells, water harvesting systems, communal points, pipelines, trucking and storage tanks).^6^^,^^12^^,^^13^

## Relevant changes

In response to the first outbreak, by the end of 2020, we were able to vaccinate 7.2 million children through two nationwide vaccination campaigns, except in Sa’ada governorate due to a ban by the authorities. In 2021, 3 800 313 children younger than 5 years had received polio vaccines. UNICEF has reported that the two campaigns reached 96% and 93% of the target population, respectively.^12^

For the second outbreak, by the end of 2022, 4 463 389 vaccines had been given to children younger than 10 years, and 1 217 423 to those younger than 5 years, an estimated 102% of the target population in the southern governorates. The ban by the authorities on vaccinations outside of health-care facilities persisted in northern governorates, despite all the efforts of WHO and UNICEF. The authorities continue to prohibit vaccination activities for polio and measles in northern governorates. An outbreak of measles has also been documented.^6^^,^^12^^,^^13^

Community volunteers reached 2 110 635 people through house-to-house visits to deliver vaccines and motivate households if there was vaccine hesitancy. A total of 11 television channels and 16 radio stations provided mass media coverage, broadcasting campaign messages and highlighting the significance of vaccination.

## Lessons learnt

Despite these renewed efforts and the successful campaign in the southern governorates, there are still political challenges and gaps in polio prevention in Yemen. The northern governorates are governed by the Houthi regime, which gained control after a coup in 2015. The official government has control of the south, but has no jurisdiction over the northern governorates which are the most conflict-affected areas. The northern authorities have continued to suspend vaccination activities in certain districts, putting these vulnerable communities at a higher risk of another polio outbreak. UNICEF and WHO still hold talks and advocate for approval to begin vaccination campaigns in conflict-affected areas. The lengthy bureaucratic authorization processes imposed by the governing authorities in the north cause delays in responding to polio outbreaks in a timely manner. Additionally, there are areas that are hard to reach, with limited access to health facilities and resources (Box 1). The only way to deliver vaccines in these areas is through primary health facilities, but no door-to-door vaccination delivery has been approved. UNICEF and WHO also continue to provide support to the primary health teams in these areas, and assist in conducting outreach programmes.

Box 1 **Summary of main lessons learnt**

- Advocacy, communication and social mobilization including grassroots community-drivenactions, were a crucial part of two successful nationwide polio vaccination campaigns.
- Lengthy bureaucratic authorization processes caused delays in responding to polio outbreaks in a timely manner.
- Limited access to health facilities and resources in conflict-affected areas, and prohibition of polio vaccination by governing authorities in some areas, created gaps in vaccination coverage.

Yemen is a water-scarce country, and the ongoing conflict has exacerbated the situation. Despite the efforts of the water, sanitation and hygiene programme to improve sanitation and ensure a safe water supply, we have underachieved in this area due to a funding gap, according to reports by UNICEF.^6^ Additionally, the sanitation infrastructure in the country is below standard and requires improvement.^6^ Activities such as hygiene promotion, and direct engagement with communities are still banned by the governing authorities in the northern governorates.^6^

Vaccination campaigns remain a crucial part of eradicating polio. Advocacy, communication and social mobilization actions emphasize the importance of a multifaceted approach, including community-driven actions, to effectively combat and control polio outbreaks. The recent polio outbreaks in Yemen serve as a reminder that in any area where vaccine coverage is low, it is only a matter of time before an outbreak occurs. Our experience also highlights the urgency of eradicating polio globally, and ensuring that every child is protected from this preventable disease. 
